# Olfaction Testing and Associated Factors among Older U.S. Farmers

**DOI:** 10.1093/geroni/igaf122.513

**Published:** 2025-12-31

**Authors:** Yaqun Yuan, Brenda Plassman, Zhehui Luo, Jayant Pinto, Christine Parks, Dale Sandler, Honglei Chen

**Affiliations:** Michigan State University, East Lansing, Michigan, United States; Duke University Medical Center, Durham, North Carolina, United States; Michigan State University, East Lansing, Michigan, United States; The University of Chicago, Chicago, Illinois, United States; National Institute of Environmental Health Sciences, Durham, North Carolina, United States; National Institute of Environmental Health Sciences, Durham, North Carolina, United States; Michigan State University, East Lansing, Michigan, United States

## Abstract

Poor olfaction is common in older adults and may signify adverse health conditions. We tested olfaction in 2545 older farmers from North Carolina and Iowa between 2020-2021, using the 12-item Brief Smell Identification Test (B-SIT). We compared their B-SIT score with perceived testing experience and examined factors that may associate with the test results, accounting for sampling design, study participation, and covariates. Overall, 37.8% of the farmers had good olfaction (B-SIT score 11-12), 34.2% moderate olfaction (9-10), and 28.0% poor olfaction (0-8). A higher B-SIT score correlated with perceived more odorants identified (Spearman’s ρ = 0.65) and perceived odor strength (Spearman’s ρ = 0.54). Older age, single marital status, North Carolina residence, and a history of asthma, acute recurrent sinus infections, and surgery of the nose or brain were associated with poor olfaction. Seasonality was associated with B-SIT results, with the highest scores in the summer (June to August) and the lowest in the Spring (March to May), with a mean difference of 0.48. Allergic or cold symptoms (e.g., running nose, sore throat, sinus pain) or farming activities (e.g., working in animal confinement areas or around wood/metal dust) in the past 24 hours were generally unrelated to poor olfaction. Although not statistically significant, those who reported loading/mixing/applying pesticides and cleaning hands or machines with gasoline in the past 24 hours had lower B-SIT scores. To our knowledge, this is the first study that characterized the olfaction testing experience in older farmers and reported factors that may associate their olfaction test results.

